# Supplementary material for: Achieving destination sustainability: How tourist-to-tourist interaction quality affects tourist loyalty?
Source: PLoS One. 2024 Aug 22;19(8):e0296944. doi: 10.1371/journal.pone.0296944 (PMC11341019; doi:10.1371/journal.pone.0296944)
Supplement: Attachment — Submitted filename: Response to Reviewers.docx [file pone.0296944.s002.docx]

IMPORTANT: PLEASE DO NOT REPLY TO THIS EMAIL

If you are unable to complete any points that are requested in this email, please explain why in the 'Enter Comments' tab of the online submission form prior to re-submitting your manuscript. This will let us quickly assess your response and send your manuscript to an Academic Editor as soon as possible.

=============================================

PONE-D-23-22635R1

Achieving Destination Sustainability: How Tourist-To-Tourist Interaction Quality Affects Tourist Loyalty?

Dr Weifeng Guo

Dear Dr. Guo,

We've checked your submission and before we can proceed, we need you to address the following issues:

1. Please include your full ethics statement in the ‘Methods’ section of your manuscript file. In your statement, please include the full name of the IRB or ethics committee who approved or waived your study, as well as whether or not you obtained informed written or verbal consent. If consent was waived for your study, please include this information in your statement as well.

**Authors’ response:** We added the full ethics statement in the “Method” section of the manuscript. To avoid exposing the authors’ identity, the university affiliation of the ethics committee was not specified in the statement. Please note that we also submitted the ethical approval statement and statement of informed consent which specify the full name of the institution that granted the IRB approval letter, along with original IRB letter and the English translation – please see the attached file.

2. Thank you for stating the following financial disclosure:

"This study is funded by China National Social Science Foundation (19XGL010)."

At this time, please address the following queries:

a) Please clarify the sources of funding (financial or material support) for your study. List the grants or organizations that supported your study, including funding received from your institution.

**Authors’ response:** This study is funded by China National Social Science Foundation (19XGL010). We have included this information in the cover letter and at the end of the manuscript.

b) State what role the funders took in the study. If the funders had no role in your study, please state: “The funders had no role in study design, data collection and analysis, decision to publish, or preparation of the manuscript.”

**Authors’ response:** The funders had no role in study design, data collection and analysis, decision to publish, or preparation of the manuscript. We have included this statement in the cover letter.

c) If any authors received a salary from any of your funders, please state which authors and which funders.

**Authors’ response:** No author received a salary from the funder. This information has been included in the cover letter.

d) If you did not receive any funding for this study, please state: “The authors received no specific funding for this work.”

**Authors’ response:** N/A.

Please include your amended statements within your cover letter; we will change the online submission form on your behalf.

**Authors’ response:** We have revised the cover letter to include the above information.

3. Thank you for stating the following in your Competing Interests section:

“The authors have no conflicts of interest in this research.”

Please complete your Competing Interests on the online submission form to state any Competing Interests. If you have no competing interests, please state "The authors have declared that no competing interests exist.", as detailed online in our guide for authors at http://journals.plos.org/plosone/s/submit-now

This information should be included in your cover letter; we will change the online submission form on your behalf.

**Authors’ response:** We have included the statement “The authors have declared that no competing interests exist.” in the cover letter.

We've returned your manuscript to your account. Please resolve these issues and resubmit your manuscript within 21 days. If you need more time, please email the journal office at plosone@plos.org. We are happy to grant extensions of up to one month past this due date. If we do not hear from you within 21 days, we will withdraw your manuscript.

Please log on to PLOS Editorial Manager at https://www.editorialmanager.com/pone/ to access your manuscript. You will find your manuscript in the 'Submissions Sent Back to Author' link under the New Submissions menu. Be sure to remove your previous manuscript file if you are uploading a new file in response to these requests. After you've made the changes requested above, please be sure to view and approve the revised PDF after rebuilding the PDF to complete the resubmission process.

We are requesting these changes to comply with the PLOS ONE submission guidelines (https://journals.plos.org/plosone/s/submission-guidelines). Please note that we won't send your manuscript for review until you have resolved the above requests.

Thank you for submitting your work to PLOS ONE and supporting our mission of Open Science.

Kind regards,

Solna Carreon Santos

PLOS ONE

__________________________________________________

In compliance with data protection regulations, you may request that we remove your personal registration details at any time. (Use the following URL: https://www.editorialmanager.com/pone/login.asp?a=r). Please contact the publication office if you have any questions.
