# Supplementary material for: Achieving destination sustainability: How tourist-to-tourist interaction quality affects tourist loyalty?
Source: PLoS One. 2024 Aug 22;19(8):e0296944. doi: 10.1371/journal.pone.0296944 (PMC11341019; doi:10.1371/journal.pone.0296944)
Supplement: Attachment — Submitted filename: Review result.docx [file pone.0296944.s003.docx]

**Review result**

**Introduction**

- Row 50 (page 3) please outline sustainable destination development with the latest referral support.
- Row 81-83 (page 4) In other words, the fulfillment of basic psychological needs plays a critical mediating role between TTI quality and tourist loyalty (preferably supported by previous research).

**Literature review and hypotheses development**

- Row 101 (page 5), literature review, and hypotheses development (please add supporting theories as the basis for this research).
- Row 133-134 (page 7), however, few scholars have investigated the relationship between TTI quality and tourist loyalty (preferably supported by previous research).
- Row 144 (page 7), Thus, TTI quality is likely to positively influence tourist loyalty (must be supported by previous research to strengthen assumptions).

**Methods**

- Row 262 (page 12) It has not revealed the sampling technique used to determine the sample.
