# Supplementary material for: Achieving destination sustainability: How tourist-to-tourist interaction quality affects tourist loyalty?
Source: PLoS One. 2024 Aug 22;19(8):e0296944. doi: 10.1371/journal.pone.0296944 (PMC11341019; doi:10.1371/journal.pone.0296944)
Supplement: Attachment — Submitted filename: Response to Reviewers.docx [file pone.0296944.s004.docx]

**Review result**

**Introduction**

- Row 50 (page 3) please outline sustainable destination development with the latest referral support.

Thanks very much for your very constructive suggestions. According to your comment, we have cited some latest article to explain the conception of sustainable tourism. We described “Sustainable development of a destination ” as “a balanced development in the economic, social, cultural, and environmental dimensions of the destination (Crabolu et al., 2023; Streimikiene et al., 2021)”.

Crabolu, G., Font, X., & Miller, G. (2023). The Hidden Power of Sustainable Tourism Indicator Schemes: Have We Been Measuring Their Effectiveness All Wrong? Journal of Travel Research

Streimikiene, D., Svagzdiene, B., Jasinskas, E., & Simanavicius, A. (2021). Sustainable tourism development and competitiveness: The systematic literature review. Sustainable Development, 29, 259–271.

- Row 81-83 (page 4) In other words, the fulfillment of basic psychological needs plays a critical mediating role between TTI quality and tourist loyalty (preferably supported by previous research).

We so appreciate for your advice. Based on you suggestion, we have used previous research to explain why the fulfillment of basic psychological needs is a feasible path linking with TTI quality and tourist loyalty in this study. Detailed modification as follow:

“given that the satisfaction of basic psychological needs has been effective transmitting mechanism behind high quality relationships and behavioral outcome (Ahn, 2019),, this study examines the mediating role the satisfaction of basic psychological needs in the influence of TTI quality on tourist loyalty”

Ahn, J. (2019). Role of harmonious and obsessive passions for autonomy, competence, and relatedness support with integrated resort experiences. Current Issues in Tourism, 23(6), 756–769.

**Literature review and hypotheses development**

- Row 101 (page 5), literature review, and hypotheses development (please add supporting theories as the basis for this research).

Thanks very much for your very constructive suggestions. We have supplemented the literature review regarding self-determination theory. Detailed content as follow:

“As a comprehensive theory of human motivation, self-determination theory emphasizes the importance of satisfying three basic psychological needs—autonomy, competence, and relatedness—for individual behavior and psychological health (Deci & Ryan, 1985, 2000). Autonomy refers to the feeling that one's actions are self-chosen; competence denotes the feeling of being effective in interactions with the environment; and relatedness indicates the feeling of being cared for and connected to others. SDT posits that a process of individuals internalizing external motivations depends on the degree to which these basic psychological needs are satisfied (Ryan & Deci, 2000). In the field of tourism research, SDT provides a robust framework for understanding and explaining tourists' motivations, behaviors, and satisfaction (e.g., Çiki & Tanriverdi, 2023; Li & Shi, 2022; Thal & Hudson, 2019). Therefore, this study applies self-determination theory to discuss the transmitting mechanism behind the impact of TTI quality on tourist loyalty from the satisfaction of basic psychological needs.”

Deci, E. L., & Ryan, R. M. (1985). Intrinsic motivation and self-determination in human behavior. Springer.

Deci, E. L., & Ryan, R. M. (2000). The “what” and “why” of goal pursuits: Human needs and the self-determination of behavior. Psychological Inquiry, 11(4), 227-268.

Ryan, R. M., & Deci, E. L. (2000). Self-determination theory and the facilitation of intrinsic motivation, social development, and well-being. American Psychologist, 55(1), 68-78.

Çiki, K. D., & Tanriverdi, H. (2023). Examining the relationships among nature-based tourists’ travel motivations, ecologically responsible attitudes and subjective well-being within the scope of self-determination theory. Current Issues in Tourism, 1–6. https://doi.org/10.1080/13683500.2023.2250509

Li, R., & Shi, Z. (2022). How does social support influence tourist-oriented citizenship behavior? A self-determination theory perspective. Frontiers in Psychology, 13. <https://doi.org/10.3389/fpsyg.2022.1043520>

Thal, K. I., & Hudson, S. (2019). A conceptual model of Wellness destination Characteristics that contribute to Psychological Well-Being. Journal of Hospitality & Tourism Research, 43(1), 41–57. <https://doi.org/10.1177/1096348017704498>

- Row 133-134 (page 7), however, few scholars have investigated the relationship between TTI quality and tourist loyalty (preferably supported by previous research).

We so appreciate for your comments. We have cited previous research to support the viewpoint that few scholars have investigated the relationship between TTI quality and tourist loyalty (Li et al., 2024)

Li, X., Yuan, Y., & Zhang, J. (2024). The influence of tourists’ emotional experiences on destination loyalty from the perspective of community economy. International Journal of Tourism Research. International Journal of Tourism Research, 26(1). <https://doi.org/10.1002/jtr.2630>

- Row 144 (page 7), Thus, TTI quality is likely to positively influence tourist loyalty (must be supported by previous research to strengthen assumptions).

Thanks your very constructive advice. We have quoted previous research to strengthen assumptions that TTI quality is likely to positively influence tourist loyalty (Zhou et al., 2023)

Zhou, G., Liu, Y., Hu, J., & Cao, X. (2023). The effect of tourist-to-tourist interaction on tourists’ behavior: The mediating effects of positive emotions and memorable tourism experiences. Journal of Hospitality and Tourism Management, 55, 161–168. <https://doi.org/10.1016/j.jhtm.2023.03.005>

**Methods**

- Row 262 (page 12) It has not revealed the sampling technique used to determine the sample.

We so appreciate for your suggestions. We have supplemented the sampling technique used by this study——convenience sample.
